# Gestational Diabetes Mellitus as an Effect Modifier of the Association of Gestational Weight Gain with Perinatal Outcomes: A Prospective Cohort Study in China

**DOI:** 10.3390/ijerph19095615

**Published:** 2022-05-05

**Authors:** Zhi-Hao Cheng, Yu-Mei Wei, Hong-Tian Li, Hong-Zhao Yu, Jian-Meng Liu, Yu-Bo Zhou

**Affiliations:** 1 Department of Epidemiology and Biostatistics, School of Public Health, Peking University, Beijing 100191, China; zhihao@pku.edu.cn (Z.-H.C.); bright@bjmu.edu.cn (H.-Z.Y.); 2 Department of Obstetrics and Gynecology, Peking University First Hospital, Beijing 100034, China; weiyumei1982@126.com; 3 National Health Commission Key Laboratory of Reproductive Health, Institute of Reproductive and Child Health, Peking University, Beijing 100191, China; liht@bjmu.edu.cn

**Keywords:** gestational weight gain, gestational diabetes mellitus, perinatal outcome, modifying effect

## Abstract

The association of gestational weight gain (GWG) with perinatal outcomes seems to differ between women with and without gestational diabetes mellitus (GDM). Whether GDM is an effect-modifier of the association has not been verified. This study aimed to assess the modifying effect of GDM on the association of GWG with perinatal outcomes. Data on 12,128 pregnant women (3013 with GDM and 9115 without GDM) were extracted from a prospective, multicenter, cohort study in China. The associations of total and trimester-specific GWG rates (GWGR) with perinatal outcomes, including small size for gestational age, large size for gestational age (LGA), preterm birth, cesarean delivery, and gestational hypertension disorders, were assessed. The modifying effect of GDM on the association was assessed on both multiplicative and additive scales, as estimated by mixed-effects logistic regression. As a result, total GWGR was associated with all of the perinatal outcomes. GDM modified the association of total GWGR with LGA and cesarean delivery on both scales (all *p* < 0.05) but did not modify the association with other outcomes. The modifying effect was observed in the third trimester but not in the first or the second trimester. Therefore, maternal GWG is associated with perinatal outcomes, and GDM modifies the association with LGA and cesarean delivery in the third trimester.

## 1. Introduction

Gestational diabetes mellitus (GDM) refers to diabetes diagnosed during pregnancy that is not clearly overt diabetes as defined by the International Federation of Gynecology and Obstetrics [1,2]. GDM is harmful to fetal growth and maternal pregnancy outcomes [3,4], and it increases the future risks of obesity and type 2 diabetes for both mother and offspring [5,6,7]. The GDM incidence estimated by the International Diabetes Federation was 13.2% worldwide in 2019 [8]; the total incidence was 14.8% in China, and it was particularly high in women of advanced age, reaching 27% [9]. Gestational weight gain (GWG) in the first trimester is associated with the occurrence of GDM [10,11], and subsequent GWG also plays a role in the prognosis of GDM [12]. GWG must be carefully monitored and managed for women with GDM.

The National Academy of Medicine (NAM; previously called the Institute of Medicine) proposed updated GWG targets in 2009, taking into account the pre-pregnancy body mass index (BMI), but not accounting for diabetic status [13]. However, the association of GWG with perinatal outcomes appeared to differ between women with and without GDM [14,15,16,17]. Meta-analyses showed a positive association of excessive GWG with a large size for gestational age (LGA), both in the general population of women and among women with GDM, but the magnitude of the association appeared to be greater in women with GDM [14,15]. Cohort studies also showed that the risks of LGA and cesarean delivery increased more rapidly with increasing GWG in women with GDM than in women without it [16,17]. Simultaneously, meta-analyses showed that insufficient GWG was associated with increased risks of a small size for gestational age (SGA) and preterm birth in the general population of women [14], but not in women with GDM [15]. These findings indicated that GDM might be an effect modifier of the association of GWG with perinatal outcomes, but no study has specifically investigated the modifying effect of GDM. In addition, GDM may occur at any time during pregnancy, but it is most likely after 24 weeks of gestation [1]; if GDM is found to modify the association, the timing at which the modifying effect exhibits also needs to be addressed. Comprehensive investigation of the modifying effect of GDM on the association of GWG with perinatal outcomes is essential to evaluating the necessity of developing GDM-specific GWG targets.

In this context, using data from a prospective, multicenter, cohort study, we aimed to directly evaluate the modifying effect of GDM on the association of GWG with perinatal outcomes and to address when the modifying effect exhibits during the pregnancy.

## 2. Materials and Methods

### 2.1. Study Participants

The prospective, multicenter University Hospital Advanced Age Pregnant (UNIHOPE) Cohort recruited pregnant women at nine tertiary hospitals in seven supercities in China between March 2017 and June 2021 [18]. The cohort consisted of a singleton pregnancy subcohort and a twin pregnancy subcohort. The present study was based on the singleton pregnancy subcohort, initially designed to recruit pregnant women of advanced age (≥35 years at delivery). At enrollment, some pregnant women of nonadvanced age (a proportion of 25%) were also recruited for potential comparisons between the two populations. The participants were enrolled and completed early pregnancy follow-up before 14 gestational weeks, completed mid-pregnancy follow-up at 24–28 gestational weeks, and late pregnancy follow-up at 32–34 gestational weeks, provided delivery information after delivery and before discharge, and completed postdelivery follow-up at 6–12 weeks postpartum.

Up to June 2021, 15,597 singleton pregnant women had concluded their pregnancies with live births. Of these, 15,492 women at eight hospitals with enrollment sizes of > 200 were initially included. Women were excluded due to (1) missing baseline characteristics or a maternal age of <20 or >50 years (n = 563); (2) missing delivery information or the offspring’s gestational age at delivery of <24 or >44 weeks, or a birth weight of <1000 or >5000 g (n = 443); (3) missing (n = 1396) or suspicious (n = 296; defined as the value exceeding median ± 3-fold of interquartile range (IQR)) maternal height, pre-pregnancy weight, pre-delivery weight, or total GWG; and (4) being diagnosed with pre-gestational diabetes (n = 662) or missing a diabetes diagnosis (n = 4). Finally, 12,128 pregnant women, including 3013 with GDM and 9115 without, were included in the analyses related to total GWG. The included and excluded women were similar in most of their characteristics except for maternal age, ethnicity, parity, and pre-pregnancy BMI (Supplementary Table S1).

For analyses of trimester-specific GWG, we further excluded those with (1) offspring with a gestational age at delivery of <28 weeks (n = 12); (2) missing (n = 1004) or suspicious (n = 230) weight or GWG at early pregnancy follow-up; and (3) missing (n = 974) or suspicious (n = 70) weight or GWG at mid-pregnancy follow-up, leaving 9838 pregnant women, including 2611 with GDM and 7227 without, in the analyses. The flowchart of the participant selection process is shown in Supplementary Figure S1.

### 2.2. Diagnosis of GDM

A 75 g oral glucose tolerance test (OGTT) at 24 to 28 gestational weeks was used to screen GDM for all of the pregnant women. Women with (1) a fasting plasma glucose ≥5.1 mmol/L, (2) an OGTT 1 h plasma glucose ≥10.0 mmol/L, or (3) an OGTT 2 h plasma glucose ≥8.5 mmol/L were diagnosed with GDM, according to the criterion of the International Association of Diabetes and Pregnancy Study Groups (IADPSG) [19].

### 2.3. GWG and Covariates

Self-reported pre-pregnancy weight was collected at enrollment. Maternal height; early, mid-, and late pregnancy weight; and pre-delivery weight were measured at the hospital. The date of the pre-delivery weight measurement was one day before delivery on average. Pre-pregnancy BMI (kg/m^2^) was calculated as pre-pregnancy weight divided by height squared, and then categorized as underweight (<18.5 kg/m^2^), normal-weight (18.5 to <25.0 kg/m^2^), or overweight/obese (≥25.0 kg/m^2^) according to the WHO BMI classification criterion [20]. Total GWG (kg) was calculated as pre-delivery weight minus pre-pregnancy weight. Total GWG rate (GWGR; kg/week) was calculated as total GWG divided by gestational weeks at delivery. First trimester GWGR was calculated as (weight at the 13th gestational week—pre-pregnancy weight)/13 weeks, second trimester GWGR as (weight at the 27th gestational week—weight at the 13th gestational week)/weeks between the two measurements, and third trimester GWGR as (pre-delivery weight − weight at the 27th gestational week)/weeks between the two measurements. If the weight was not measured at the 13th or 27th gestational week, a linear interpolation method was used to estimate the weight at the 13th or 27th gestational week [21]. Covariates, including maternal age, ethnicity, parity, conception mode, and pre-pregnancy smoking status, were collected using a standard questionnaire by trained obstetricians or nurses.

### 2.4. Perinatal Outcomes

The perinatal outcomes of interest were SGA, LGA, preterm birth, cesarean delivery, and gestational hypertensive disorders (GHDs). SGA and LGA were defined as birth weight <10th and >90th percentiles of birth weight for gestational age, respectively, according to the Chinese gestational age- and sex-specific birth weight standards [22]. Preterm birth was defined as birth with gestational age at delivery < 37 weeks. GHDs were defined as systolic blood pressure > 140 mmHg or diastolic blood pressure > 90 mmHg that appeared or was first recognized after 20 gestational weeks.

### 2.5. Statistical Analyses

Maternal characteristics are presented for women with and without GDM, respectively. Continuous variables are presented as means ± standard deviations (SDs) if normally distributed, medians (IQRs) if skewed, and categorical variables as frequencies (%). The difference between the two groups was tested via a Student’s *t*-test or Wilcoxon rank-sum test for continuous variables and a chi-square test for categorical variables.

To analyze the relationship between total GWGR and perinatal outcomes, mixed-effects logistic regression was used to estimate the adjusted incidences of the perinatal outcomes in each quintile of total GWGR, and to estimate the adjusted odds ratio (AOR) of the perinatal outcomes for SD of the total GWGR increase for women both with and without GDM. Because of the identified nonlinear relationship between total GWGR and preterm birth according to the Wald test, AORs of preterm birth were separately estimated before and after a breakpoint of total GWGR. The location of the breakpoint was determined by grid search method and the Akaike information criterion. Adjusted covariates included pre-pregnancy BMI, maternal age, ethnicity, parity, conception mode, and smoking status. Center effects were adjusted by including appropriate random effects of centers.

The modifying effect of GDM on the association of total GWGR with the perinatal outcomes was assessed on both multiplicative and additive scales. The multiplicative interaction (INT_M_) was measured by exponential coefficient of the interaction term, and the additive interaction by relative excess risk due to interaction (RERI) [23]. To verify the interactions in a younger population group, the analysis was performed among the women who were younger than the median age of the participants (36 years). To clarify the interactions in women with different pre-pregnancy BMIs, a stratified analysis by pre-pregnancy BMI was performed. For the outcomes in which the modifying effect of GDM was observed in total GWGR analyses, the multiplicative and additive interactions between trimester-specific GWGR and GDM were further assessed.

Statistical analyses were performed using R software (version: 4.0; R Foundation for Statistical Computing, Vienna, Austria) with a two-sided test. A *p* value < 0.05 was considered statistically significant.

## 3. Results

### 3.1. Characteristics of Study Participants

Maternal characteristics for women with and without GDM are presented in Table 1. The total GWGR was lower in women with GDM than in women without it (0.30 ± 0.13 kg/week vs. 0.35 ± 0.13 kg/week). The first trimester GWGR was higher (0.15 ± 0.16 vs. 0.14 ± 0.17 kg/week), the second trimester (0.38 ± 0.19 vs. 0.43 ± 0.19 kg/week) and third trimester (0.35 ± 0.32 vs. 0.48 ± 0.32 kg/week) GWGRs were lower in women with GDM than in women without. As compared with women without GDM, women with GDM were more likely to have a higher maternal age and a higher pre-pregnancy BMI, to conceive via assisted reproductive technology, and to deliver a neonate with a lower birth weight and at an earlier gestational age (*p* < 0.01 for all the comparisons). Among women with GDM, 15.3% were treated with insulin, and the rest were only treated with lifestyle interventions, including diet therapy and/or exercise.

### 3.2. Incidences of Perinatal Outcomes with Total GWGR

**Figure 1:**
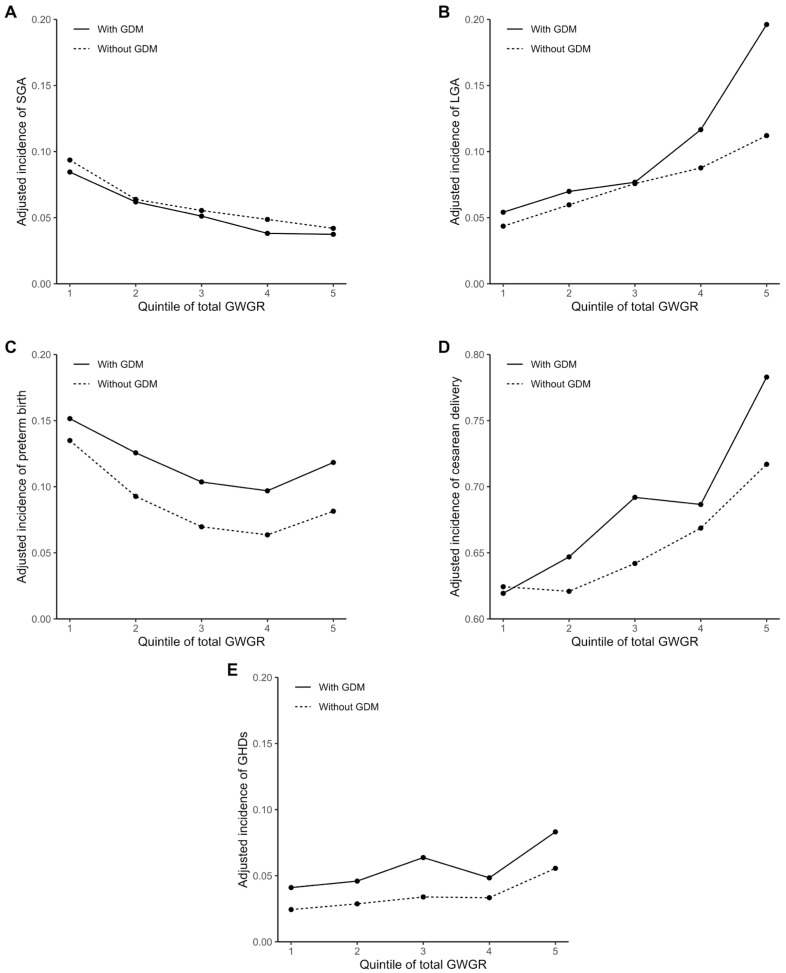
Adjusted incidences of perinatal outcomes with total GWGR: (**A**) adjusted incidence of small size for gestational age (SGA); (**B**) adjusted incidence of large size for gestational age (LGA); (**C**) adjusted incidence of preterm birth; (**D**) adjusted incidence of cesarean delivery; (**E**) and adjusted incidence of gestational hypertension disorders (GHDs). Participants were subdivided into five groups according to the quintile of total GWGR. The adjusted incidences of the perinatal outcomes were estimated by mixed-effects logistic regression with the pre-pregnancy BMI, maternal age, ethnicity, parity, conception mode, and smoking status adjusted and fixed, respectively, at 22.0 kg/m^2^, ≥35 and <40 years, Han ethnicity, multipara, natural conception, and nonsmoking, for both women with and without GDM (mean or most frequent category of the data).

The adjusted incidences of the perinatal outcomes with a total GWGR change are shown in Figure 1. Overall, the incidences of LGA, preterm birth, cesarean delivery, and GHDs were higher in women with GDM than in women without, given the same total GWGR. The incidences of LGA, cesarean delivery, and GHDs increased, and the incidence of SGA decreased with increasing total GWGR. A U-shaped relationship was observed between total GWGR and preterm birth. In addition, the incidences of LGA and cesarean delivery appeared to increase more rapidly with increasing total GWGR in women with GDM than in women without.

### 3.3. Modifying Effect of GDM on Association of Total GWGR with Perinatal Outcomes

The modifying effect of GDM on the association of total GWGR with perinatal outcomes is shown in Table 2. GDM modified the association of total GWGR with LGA (INT_M_ = 1.24, 95% CI: 1.08–1.43; RERI = 0.39, 95% CI: 0.13–0.65) and cesarean delivery (INT_M_ = 1.10, 95% CI: 1.001–1.22; RERI = 0.15, 95% CI: 0.01–0.29). For SD of total GWGR (0.13 kg/week) increase, the odds of LGA increased by 76% in women with GDM and 41% in women without (AOR = 1.76, 95% CI: 1.54–2.00 vs. AOR = 1.41, 95% CI: 1.30–1.53), and the corresponding odds of cesarean delivery increased by 29% and 21% (AOR = 1.29, 95% CI: 1.17–1.41 vs. AOR = 1.21, 95% CI: 1.15–1.27). GWG was associated with the odds of SGA, preterm birth, and GHDs, but the modifying effect was not observed for these associations. Similar results remained when analysis was restricted to women younger than the median age of the participants (Supplementary Table S2).

**Figure 2:**
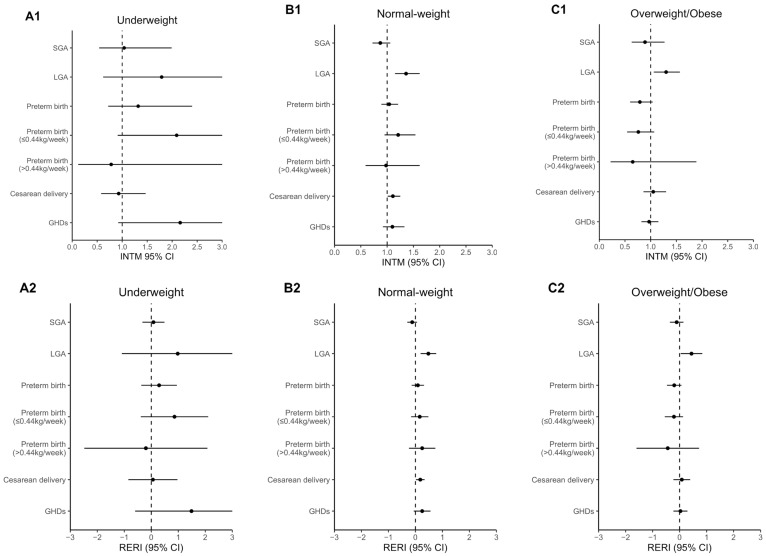
Modifying effect of GDM on association of total GWGR with perinatal outcomes by pre-pregnancy BMI categories: (**A1**) multiplicative interaction between total GWGR and GDM in underweight women; (**A2**) additive interaction in underweight women; (**B1**) multiplicative interaction in normal-weight women; (**B2**) additive interaction in normal-weight women; (**C1**) multiplicative interaction in overweight/obese women; (**C2**) additive interaction in overweight/obese women. Mixed-effects logistic regression was used to estimate the INT_M_ and RERI, with adjustment of the covariates of maternal age, ethnicity, parity, conception mode, and smoking status. The parts of the confidence interval exceeding 0 to 3 for INT_M_ and −3 to 3 for RERI are not shown in the figure.

The estimates of the modifying effect and the association by pre-pregnancy BMI categories are shown in Figure 2 and Supplementary Table S3, respectively. The modifying effect of GDM on the association of total GWGR with LGA was significant in normal-weight (INT_M_ = 1.36, 95% CI: 1.15–1.62; RERI = 0.48, 95% CI: 0.19–0.77) and overweight/obese women (INT_M_ = 1.30, 95% CI: 1.06–1.57; RERI = 0.44, 95% CI: 0.04–0.84), but not in underweight women. The modifying effect on the association with cesarean delivery was only significant in normal-weight women (INT_M_ = 1.11, 95% CI: 1.01–1.25; RERI = 0.18, 95% CI: 0.02–0.34).

### 3.4. Modifying Effect of GDM on Association of Trimester-Specific GWGR with Perinatal Outcomes

The modifying effect of GDM on the association of trimester-specific GWGR with LGA and cesarean delivery is shown in Table 3. GDM modified the association of third trimester GWGR with LGA (INT_M_ = 1.39, 95% CI: 1.19–1.62; RERI = 0.46, 95% CI: 0.22–0.71) and cesarean delivery (INT_M_ = 1.15, 95% CI: 1.01–1.31; RERI = 0.19, 95% CI: 0.02–0.36). For SD of third trimester GWGR (0.32 kg/week) increase, the odds of LGA increased by 52% in women with GDM and by 9% in women without (AOR = 1.52, 95% CI: 1.33–1.75 vs. AOR = 1.09, 95% CI: 1.002–1.19), and the corresponding odds of cesarean delivery increased by 28% and 15% (AOR = 1.28, 95% CI: 1.14–1.44 vs. AOR = 1.15, 95% CI: 1.08–1.22). The modifying effect of GDM on the association of first or second trimester GWGR with LGA or cesarean delivery was not observed.

## 4. Discussion

In this large, prospective, multicenter, cohort study, we first assessed the modifying effect of GDM on the association of GWG with the common perinatal outcomes on both multiplicative and additive scales. We found that GDM modified the association of GWGR with LGA and cesarean delivery on both scales, but it did not modify the association with SGA, preterm birth, or GHDs. Trimester-specific analyses further showed that the modifying effect for LGA and cesarean delivery exhibited in the third trimester.

In the present study, we found not only the multiplicative interaction, also called the “statistical interaction”, between total GWGR and GDM for LGA, but also the additive interaction, also called the “biological interaction”, and considered to be more indicative of the underlying causal mechanism [24]. Specifically, for SD of total GWGR increase, the relative risk of LGA increased by 24% and 0.39 more in women with GDM than in women without on the multiplicative and additive scales (INT_M_ = 1.24; RERI = 0.39), respectively [25]. The modifying effect was presented in normal-weight and overweight/obese women, but not in underweight women, which may reflect that the modifying effect differs across pre-pregnancy BMI, but it may also be due to the small sample size of underweight women (n = 995). The modifying effect was further found to exhibit in the third trimester, indicating the different effect of trimester-specific GWG between women with and without GDM. We found the strongest GWG effect on LGA in the second trimester for women without GDM, in line with previous findings that the second trimester was a critical window of GWG effect on fetal growth [26,27]. In contrast, the effect of trimester-specific GWG on fetal growth has been less investigated for women with GDM [28], and we found that the GWG effect on LGA in the third trimester appeared stronger than that in the second, indicating that the third trimester might also be a critical window for GWG effects on fetal growth for women with GDM. The modifying effect of GDM for LGA might be biologically explained. Excessive GWG could worsen glycemic control and lead to hyperglycemia in women with GDM [29], which is less likely to occur in women without GDM. Hyperglycemia could result in a higher birth weight and an increased risk of LGA [30].

For cesarean delivery, we also observed the modifying effect of GDM on both scales, which exhibited in the third trimester. For SD of total GWGR increase, the relative risk of cesarean delivery increased by 10% and 0.15 more in women with GDM than in women without on the multiplicative and additive scales (INT_M_ = 1.10; RERI = 0.15), respectively. The hypersensitivity to GWG regarding cesarean delivery for women with GDM might also be related to the relationship between GWG and glycemia. Higher glycemia is associated with GHDs as well as LGA [31], which are common indications for cesarean delivery [32,33]. In addition, a higher glycemia could lead to vaginal dysbiosis, which increases the risk of various vaginal infections, leading to a higher risk of cesarean delivery [34,35,36].

We did not find the modifying effect of GDM on the association of GWGR with SGA or preterm birth although the association appeared to somewhat differ between women with and without GDM in previous studies [14,15,28,37,38,39]. For example, a meta-analysis of the general population of women reported a significant association of insufficient GWG with SGA and preterm birth [14], but in women with GDM, combining five studies for SGA (n = 5245; RR = 1.40, 95% CI: 0.86–2.27) and four studies for preterm birth (n = 3142; RR = 1.01, 95% CI: 0.76–1.34) [15], a significant association was not found. In a post hoc analysis of our study, we found that the insufficient GWG according to the NAM targets was associated with increased risks of SGA (AOR = 1.61, 95% CI: 1.20–2.16 and AOR = 1.43, 95% CI: 1.19–1.71) and preterm birth (AOR = 1.57, 95% CI: 1.22–2.02 and AOR = 1.68, 95% CI: 1.42–1.99) in both women with and without GDM. Besides the difference in maternal characteristics among the studies, the large sample size of GDM women in our study (n = 3013) somewhat guaranteed a sufficient statistical power to detect a statistical difference.

Given that the risks of LGA and cesarean delivery increased more rapidly with GWG increase in women with GDM than in women without in our study, a lower GWG for women with GDM than for women without might be reasonable. Furthermore, previous studies have found improvements from a lower GWG than the NAM targets on perinatal outcomes in overweight/obese women with GDM [40,41,42,43], but the impact of a lower GWG in normal-weight or underweight women is unknown. Our findings, that the modifying effect also occurred in normal-weight women, indicated that the normal-weight women with GDM may also benefit from a lower GWG.

Our study has some limitations. First, most participants were women of advanced age (a proportion of 81%), which might jeopardize the generalization of the findings to a younger population although the modifying effect of GDM persisted among the women younger than the median age of our participants. Second, glycemic data was not available, preventing us from exploring the role of glycemia in the modifying effect. Third, GDM was diagnosed at 24–28 gestational weeks in the study; the time of GDM diagnosis and the interventions after diagnosis may influence the findings on the timing at which the modifying effect exhibits. Fourth, pre-pregnancy weight was self-reported and might suffer from recall bias, despite the high amount of agreement between self-reported and measured weight [44,45].

## 5. Conclusions

In this prospective cohort study, we found that GDM modified the association of GWG with LGA and cesarean delivery, and this modifying effect was only observed in the third trimester. A higher GWG during the third trimester confers greater risks of LGA and cesarean delivery on women with GDM than on women without, which presented not only in women who were overweight/obese before pregnancy but also in those who were normal-weight. These findings indicated that the optimal GWG might be lower for women with GDM than for women without GDM, especially in the third trimester. More large-cohort studies are expected to determine the optimal GWG range for women with GDM. The role of blood glucose in the modifying effect also needs to be ascertained.

## Figures and Tables

**Table 1:** Baseline characteristics of study participants.

	Without GDM (n = 9115)	With GDM (n = 3013)	*p* Value ^a^
Maternal age, year	36.0 (35.0–38.0)	37.0 (36.0–39.0)	<0.001
<35	1966 (21.6)	312 (10.4)	<0.001
≥35 and <40	5829 (63.9)	2047 (67.9)	
≥40	1320 (14.5)	654 (21.7)	
Han ethnicity	8780 (96.3)	2911 (96.6)	0.459
Multipara	5296 (58.1)	1754 (58.2)	0.913
Conception by ART	1254 (13.8)	590 (19.6)	<0.001
Smoking ^b^	142 (1.6)	42 (1.4)	0.523
Pre-pregnancy BMI, kg/m^2^	21.8 ± 2.8	22.8 ± 3.0	<0.001
Total GWG, kg	13.5 ± 5.0	11.4 ± 5.1	<0.001
GWGR, kg/week			
Total	0.35 ± 0.13	0.30 ± 0.13	<0.001
First trimester ^c^	0.14 ± 0.17	0.15 ± 0.16	0.004
Second trimester ^c^	0.43 ± 0.19	0.38 ± 0.19	<0.001
Third trimester ^c^	0.48 ± 0.32	0.35 ± 0.32	<0.001
Birth weight, g	3262.0 ± 482.0	3231.0 ± 511.0	0.002
Gestational age, week	39.0 (38.3–39.9)	38.6 (38.0–39.6)	<0.001

Abbreviations: GDM, gestational diabetes mellitus; ART, assisted reproductive technology; BMI, body mass index; GWG, gestational weight gain; and GWGR, gestational weight gain rate. Data are expressed as means ± standard deviations, medians (interquartile ranges), or frequencies (%). ^a^ A Student’s *t*-test or Wilcoxon rank-sum test for continuous variables and a chi-square test for categorical variables were used to examine the difference between the two groups. ^b^ Smoking during the 6 months before pregnancy to the enrollment. ^c^ The numbers of women with and without GDM for calculating the trimester-specific GWGR were 2611 and 7227, respectively.

**Table 2:** Modifying effect of GDM on association of total GWGR with perinatal outcomes.

Outcome	AOR (95% CI)			INT_M_ (95% CI)	RERI (95% CI)
	All Women (n = 12,128)	Without GDM (n = 9115)	With GDM (n = 3013)		
SGA ^a^	0.75 (0.70–0.81)	0.78 (0.71–0.85)	0.70 (0.61–0.82)	0.90 (0.77–1.05)	−0.09 (−0.21–0.02)
LGA ^b^	1.50 (1.40–1.60)	1.41 (1.30–1.53)	1.76 (1.54–2.00)	1.24 (1.08–1.43)	0.39 (0.13–0.65)
Preterm birth ^c^	1.07 (0.98–1.14)	1.07 (0.99–1.16)	1.08 (0.97–1.21)	1.01 (0.89–1.14)	0.03 (−0.14–0.20)
GWGR ≤ 0.44kg/week ^d^	0.87 (0.76–0.99)	0.84 (0.71–0.99)	0.90 (0.72–1.12)	1.01 (0.78–1.31)	−0.01 (−0.23–0.20)
GWGR > 0.44kg/week ^e^	1.40 (1.24–1.57)	1.42 (1.24–1.63)	1.38 (1.09–1.75)	0.95 (0.73–1.24)	0.03 (−0.16–0.23)
Cesarean delivery ^f^	1.23 (1.18–1.28)	1.21 (1.15–1.27)	1.29 (1.17–1.41)	1.10 (1.001–1.22)	0.15 (0.01–0.29)
GHDs ^g^	1.32 (1.22–1.42)	1.32 (1.20–1.45)	1.29 (1.14–1.47)	1.01 (0.88–1.18)	0.15 (−0.11–0.42)

The breakpoint of total GWGR for the association with preterm birth was at 0.44 kg/week. Mixed-effects logistic regression was used to estimate the adjusted odds ratio (AOR), multiplicative interaction (INT_M_), relative excess risk due to interaction (RERI), and their confidence intervals (CIs), with adjustment of the covariates of pre-pregnancy BMI, maternal age, ethnicity, parity, conception mode, and smoking status in women with GDM and women without, further adjustment of GDM status (with or without GDM) in all women. A Wald test was used to examine a potential nonlinear association of total GWGR with perinatal outcomes in all women: ^a^ *p* = 0.98; ^b^ *p* = 0.47; ^c^ *p* < 0.001; ^d^ *p* = 0.93; ^e^ *p* = 0.27; ^f^ *p* = 0.06; and ^g^ *p* = 0.16.

**Table 3:** Modifying effect of GDM on association of trimester-specific GWGR with LGA and cesarean delivery.

Outcome	Trimester	AOR (95% CI)			INT_M_ (95% CI)	RERI (95% CI)
		All Women (n = 9838)	Without GDM (n = 7227)	With GDM (n = 2611)		
LGA	First	1.15 (1.07–1.24)	1.15 (1.05–1.25)	1.17 (1.01–1.35)	1.05 (0.90–1.24)	0.05 (−0.13–0.23)
	Second	1.50 (1.39–1.62)	1.53 (1.40–1.67)	1.43 (1.24–1.64)	0.97 (0.83–1.13)	−0.05 (−0.28–0.19)
	Third	1.20 (1.12–1.29)	1.09 (1.002–1.19)	1.52 (1.33–1.75)	1.39 (1.19–1.62)	0.46 (0.22–0.71)
Cesarean delivery	First	1.07 (1.02–1.13)	1.08 (1.02–1.14)	1.06 (1.001–1.14)	0.98 (0.88–1.08)	−0.02 (−0.14–0.09)
	Second	1.22 (1.15–1.30)	1.21 (1.13–1.30)	1.24 (1.11–1.39)	1.03 (0.91–1.18)	0.04 (−0.13–0.20)
	Third	1.18 (1.12–1.25)	1.15 (1.08–1.22)	1.28 (1.14–1.44)	1.15 (1.01–1.31)	0.19 (0.02–0.36)

Mixed-effects logistic regression was used to estimate the AOR, INT_M_, RERI, and their CIs, with adjustment of the covariates of pre-pregnancy BMI, maternal age, ethnicity, parity, conception mode, smoking status, and previous trimester GWGRs in women with GDM and women without, further adjustment of GDM status (with or without GDM) in all women.

## Data Availability

The data presented in this study are available upon request from the corresponding author.

## Supplementary Materials

The following supporting information can be downloaded at: https://www.mdpi.com/article/10.3390/ijerph19095615/s1: Figure S1: Study participant selection process; Table S1: Comparison of characteristics between included and excluded participants; Table S2: Modifying effect of GDM on association of total GWGR with perinatal outcomes among women younger than the median age of participants; Table S3: Association of total GWGR with perinatal outcomes in women with and without GDM by pre-pregnancy BMI categories.
