# Supplementary material for: Gestational Diabetes Mellitus as an Effect Modifier of the Association of Gestational Weight Gain with Perinatal Outcomes: A Prospective Cohort Study in China
Source: Int J Environ Res Public Health. 2022 May 5;19(9):5615. doi: 10.3390/ijerph19095615 (PMC9101455; doi:10.3390/ijerph19095615)
Supplement: Supplementary file 1 [file ijerph-19-05615-s001.zip › ijerph-1617480-supplementary.pdf]

Supplement for "Gestational Diabetes Mellitus as An Effect Modifier of Association of Gestational Weight Gain with Perinatal Outcomes: A Prospective Cohort Study in China"

Supplemental Tables

Table S1. Comparison of characteristics between included and excluded participants.

Table S2. Modifying effect of GDM on association of total GWGR with perinatal outcomes among women younger than the median age of participants.

Table S3. Association of total GWGR with perinatal outcomes in women with and without GDM by pre-pregnancy BMI categories.

Supplemental Figures

Figure S1. Study participant selection process.

Table S1. Comparison of characteristics between included and excluded participants.

	Included (n = 12128)	Excluded (n = 2538) [a]	p Value [b]
Maternal age, year	36.0 (35.0 to 38.0)	36.0 (31.0 to 38.0) [n = 2521]	< 0.001
Han ethnicity	11691 (96.4)	2331 (93.4) [n = 2495]	< 0.001
Multipara	7050 (58.1)	1094 (43.1) [n = 2538]	< 0.001
Conception by ART	1844 (15.2)	323 (14.1) [n = 2288]	0.06
Smoking [c]	184 (1.5)	37 (1.7) [n = 2159]	0.50
Pre-pregnancy BMI, kg/m^2	22.0 ± 2.9	22.3 ± 3.6 [n = 2018]	0.02
Total GWG, kg	13.0 ± 5.1	13.2 ± 5.1 [n = 572]	0.22
Total GWGR, kg/week	0.34 ± 0.13	0.35 ± 0.13 [n = 444]	0.10
Birth weight, g	3254.5 ± 489.2	3270.0 ± 504.0 [n = 2344]	0.22
Gestational age, week	39.0 (38.3 to 39.7)	39.1 (38.1 to 39.9) [n = 2346]	0.25

Abbreviations: ART, assisted reproductive technology; BMI, body mass index; GWG, gestational weight gain; GWGR, gestational weight gain rate. Data are expressed as means ± SDs or medians (IQRs) or frequencies (%) [numbers of available participants in current variable]. [a] Of the initial 15,492 participants, 3,013 women with GDM and 9,115 women without were finally included in the analyses; of the remaining 3,364 pregnant women, 785 were diagnosed with pre-gestational diabetes, 41 missed diabetes diagnosis, and the remaining 439 women with and 2,099 women without GDM excluded due to missing or suspicious values were listed in the table (n = 2,538). [b] Student's t-test or Wilcoxon rank-sum test for continuous variables and Chi-squared test for categorical variables were used to examine the difference between the two groups. [c] Smoking during the six months before pregnancy to the enrollment.

Table S2. Modifying effect of GDM on association of total GWGR with perinatal outcomes among women younger than the median age of participants.

Outcome	AOR (95% CI)			INT_M (95% CI)	RERI (95% CI)
	All women (n = 12128)	Without GDM (n = 4823)	With GDM (n = 1260)		
SGA	0.78 (0.71 to 0.87)	0.80 (0.72 to 0.90)	0.73 (0.59 to 0.91)	0.93 (0.74 to 1.17)	-0.08 (-0.26 to 0.10)
LGA	1.42 (1.29 to 1.56)	1.33 (1.20 to 1.49)	1.74 (1.43 to 2.12)	1.34 (1.09 to 1.65)	0.50 (0.11 to 0.90)
Preterm birth	1.06 (0.97 to 1.17)	1.07 (0.96 to 1.20)	1.04 (0.86 to 1.24)	1.02 (0.84 to 1.26)	0.06 (-0.23 to 0.34)
GWGR≤0.44kg/week	0.90 (0.78 to 1.04)	0.87 (0.73 to 1.03)	0.97 (0.75 to 1.27)	1.16 (0.86 to 1.57)	0.14 (-0.26 to 0.54)
GWGR>0.44 kg/week	1.46 (1.21 to 1.77)	1.49 (1.20 to 1.84)	1.44 (0.95 to 2.19)	1.00 (0.64 to 1.56)	0.13 (-0.47 to 0.72)
Cesarean delivery	1.22 (1.15 to 1.29)	1.19 (1.11 to 1.27)	1.36 (1.20 to 1.55)	1.12 (1.05 to 1.20)	0.27 (0.04 to 0.50)
GHDs	1.34 (1.20 to 1.50)	1.29 (1.12 to 1.47)	1.49 (1.20 to 1.85)	1.20 (0.94 to 1.53)	0.37 (-0.08 to 0.82)

Mixed-effects logistic regression was used to estimate the adjusted odds ratio (AOR), multiplicative interaction (INT_M), and relative excess risk due to interaction (RERI), and their confidence intervals (CIs), with adjustment of the covariates of pre-pregnancy BMI, ethnicity, parity, conception mode, and smoking status in women with GDM and women without, further adjustment of GDM status (with or without GDM) in all women. Abbreviations: SGA, small size for gestational age; LGA, large size for gestational age; GHDs, gestational hypertension disorders.

Table S3. Association of total GWGR with perinatal outcomes in women with and without GDM by pre-pregnancy BMI categories.

Outcome	AOR (95% CI)		
	**All women**	**Without GDM**	**With GDM**
Underweight women	n = 995	n = 832	n = 163
SGA	0.71 (0.56 to 0.90)	0.71 (0.55 to 0.92)	0.74 (0.37 to 1.47)
LGA	1.80 (1.22 to 2.66)	1.68 (1.09 to 2.59)	3.34 (1.18 to 9.51)
Preterm birth	1.07 (0.84 to 1.37)	1.01 (0.77 to 1.34)	1.37 (0.79 to 2.40)
GWGR ≤ 0.44 kg/week	0.93 (0.67 to 1.30)	0.80 (0.55 to 1.17)	1.67 (0.78 to 3.58)
GWGR > 0.44 kg/week	1.67 (0.91 to 3.07)	1.60 (0.82 to 3.11)	0.12 (0.01 to 13.26)
Cesarean delivery	1.32 (1.12 to 1.56)	1.35 (1.13 to 1.61)	1.29 (0.84 to 2.00)
GHDs	1.01 (0.69 to 1.48)	0.84 (0.53 to 1.33)	1.69 (0.83 to 3.43)
Normal-weight women	n = 9274	n = 7090	n = 2184
SGA	0.77 (0.71 to 0.84)	0.80 (0.72 to 0.88)	0.70 (0.59 to 0.84)
LGA	1.39 (1.28 to 1.50)	1.28 (1.17 to 1.41)	1.77 (1.51 to 2.07)
Preterm birth	1.07 (1.00 to 1.15)	1.06 (0.97 to 1.15)	1.14 (1.00 to 1.31)
GWGR ≤ 0.44 kg/week	0.87 (0.77 to 0.97)	0.81 (0.70 to 0.93)	1.02 (0.83 to 1.26)
GWGR > 0.44 kg/week	1.67 (1.34 to 2.08)	1.68 (1.31 to 2.15)	1.75 (1.11 to 2.77)
Cesarean delivery	1.17 (1.11 to 1.23)	1.15 (1.08 to 1.21)	1.25 (1.13 to 1.38)
GHDs	1.24 (1.13 to 1.35)	1.20 (1.08 to 1.34)	1.30 (1.12 to 1.51)
Overweight/Obese women	n = 1859	n = 1193	n = 666
SGA	0.83 (0.70 to 0.98)	0.86 (0.69 to 1.08)	0.79 (0.60 to 1.04)
LGA	1.41 (1.23 to 1.61)	1.31 (1.11 to 1.52)	1.70 (1.37 to 2.10)
Preterm birth	0.99 (0.87 to 1.13)	1.08 (0.92 to 1.26)	0.86 (0.68 to 1.08)
GWGR ≤ 0.44 kg/week	0.94 (0.80 to 1.11)	1.04 (0.84 to 1.28)	0.80 (0.61 to 1.04)
GWGR > 0.44 kg/week	1.11 (0.72 to 1.70)	1.20 (0.74 to 1.95)	0.87 (0.32 to 2.37)
Cesarean delivery	1.22 (1.11 to 1.35)	1.21 (1.07 to 1.36)	1.28 (1.08 to 1.53)
GHDs	1.18 (1.09 to 1.28)	1.20 (1.08 to 1.33)	1.15 (1.00 to 1.33)

The pre-pregnancy BMI were <18.5 kg/m^2 for underweight women, 18.5 to <25 kg/m^2 for normal-weight women, and ≥25 kg/m^2 for overweight/obese women. Mixed-effects logistic regression was used to estimate the AOR and 95% CI with adjustment of the covariates of maternal age, ethnicity, parity, conception mode, and smoking status in women with GDM and women without, further adjustment of GDM status (with or without GDM) in all women.

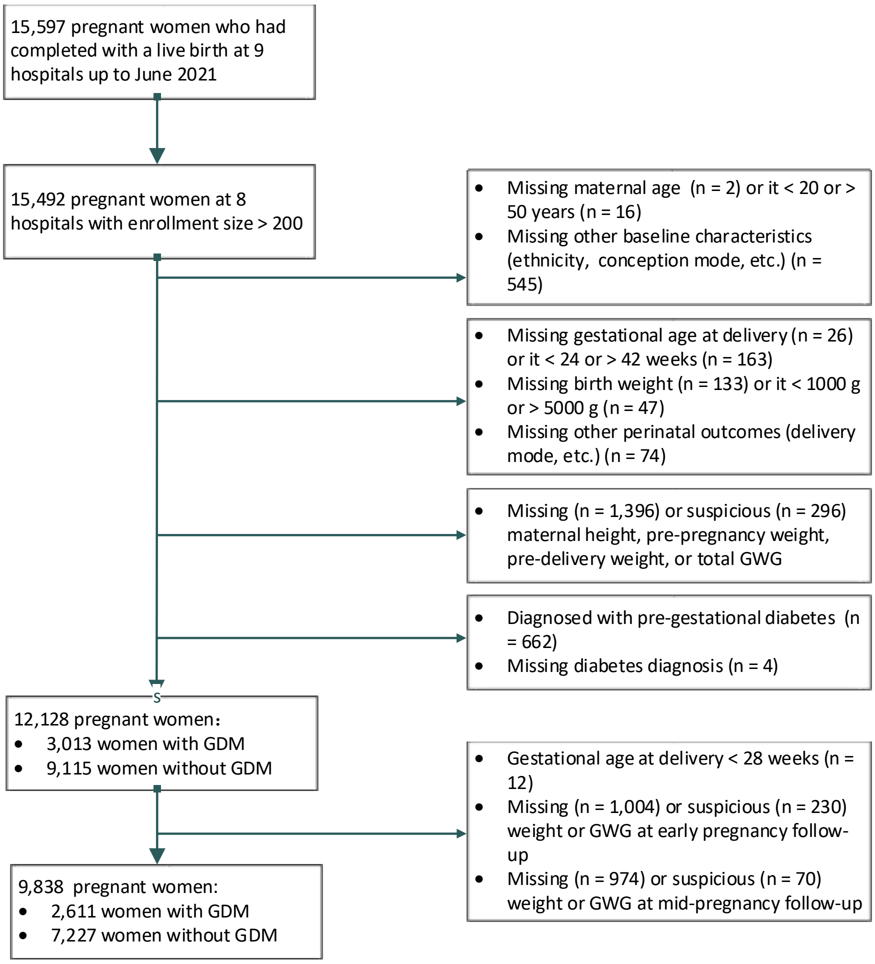

Figure S1. Study participant selection process.
